# Gentle Mechanical Skin Stimulation Inhibits Micturition Contractions via the Spinal Opioidergic System and by Decreasing Both Ascending and Descending Transmissions of the Micturition Reflex in the Spinal Cord

**DOI:** 10.1371/journal.pone.0135185

**Published:** 2015-08-07

**Authors:** Harumi Hotta, Nobuhiro Watanabe

**Affiliations:** Department of Autonomic Neuroscience, Tokyo Metropolitan Institute of Gerontology, Tokyo, Japan; University of California, Los Angeles, UNITED STATES

## Abstract

Recently, we found that gentle mechanical skin stimulation inhibits the micturition reflex in anesthetized rats. However, the central mechanisms underlying this inhibition have not been determined. This study aimed to clarify the central neural mechanisms underlying this inhibitory effect. In urethane-anesthetized rats, cutaneous stimuli were applied for 1 min to the skin of the perineum using an elastic polymer roller with a smooth, soft surface. Inhibition of rhythmic micturition contractions by perineal stimulation was abolished by naloxone, an antagonist of opioidergic receptors, administered into the intrathecal space of the lumbosacral spinal cord at doses of 2–20 μg but was not affected by the same doses of naloxone administered into the subarachnoid space of the cisterna magna. Next, we examined whether perineal rolling stimulation inhibited the descending and ascending limbs of the micturition reflex. Perineal rolling stimulation inhibited bladder contractions induced by electrical stimulation of the pontine micturition center (PMC) or the descending tract of the micturition reflex pathway. It also inhibited the bladder distension-induced increase in the blood flow of the dorsal cord at L5–S1, reflecting the neural activity of this area, as well as pelvic afferent-evoked field potentials in the dorsal commissure at the lumbosacral level; these areas contain long ascending neurons to the PMC. Neuronal activities in this center were also inhibited by the rolling stimulation. These results suggest that the perineal rolling stimulation activates the spinal opioidergic system and inhibits both ascending and descending transmissions of the micturition reflex pathway in the spinal cord. These inhibitions would lead to the shutting down of positive feedback between the bladder and the PMC, resulting in inhibition of the micturition reflex. Based on the central neural mechanisms we show here, gentle perineal stimulation may be applicable to several different types of overactive bladder.

## Introduction

Somatic stimulation has been used for the treatment of various autonomic nervous system dysfunctions such as voiding and storage dysfunctions of the urinary bladder. For example, acupuncture or transcutaneous electrical stimulation has successfully been used to treat an overactive bladder [1, 2]. However, the mechanisms underlying this effect are not fully clarified.

Mechanisms underlying somatic modulation of bladder functions have been studied in anesthetized animals [3, 4]. When the bladder is full, rhythmic micturition contractions (RMCs) emerge because of burst discharges of the pelvic nerve innervating the bladder. Various kinds of noxious somatic stimuli, particularly those applied to the perineal area, consistently produce a decrease in frequency of RMCs in both male and female animals [5–7]. Following somatic stimulation, parasympathetic pelvic efferent nerve activity, which controls bladder contraction, is inhibited, whereas sympathetic hypogastric nerve activity is sometimes facilitated during perineal stimulation. The inhibition of RMCs by perineal stimulation is not affected by total destruction of the hypogastric nerve branches, indicating that parasympathetic nerves, but not sympathetic nerves, are involved in this effect as an efferent path [3].

In the case of non-noxious cutaneous mechanical stimulations, we have recently shown that the effect depends on materials in contact with the skin [8, 9]. Briefly, gentle stimulation of the surface of the perineal skin with a soft roller (made of elastic polymer) inhibited RMCs in anesthetized rats. Stimulation consisted of just contacting the skin and moving the roller slowly. Stimulation for 1 min inhibited bladder contractions during and after stimulation for several minutes. Stimuli applied to other areas such as the hind limb or abdomen also produced significant inhibition, but perineal stimulation produced the most consistent inhibition. The inhibitory effect of perineal stimulation was almost abolished by an intravenous administration of naloxone, an opioid receptor antagonist. Perineal stimulation excited low-threshold mechanoreceptive Aβ, Aδ, and C afferent fibers at mean frequencies of 2, 3, and 8 Hz, respectively. Reflex discharges in the pelvic efferent nerve, induced by appropriate isotonic bladder distensions, were decreased by approximately 50% by perineal stimuli. These results suggest that low-frequency excitation of cutaneous mechanoreceptive myelinated and unmyelinated fibers inhibits a vesico-pelvic parasympathetic reflex, mainly via the release of opioids, leading to long-lasting inhibition of micturition contraction [8].

The inhibition of RMCs by perineal rolling stimulation must occur at some site(s) of the micturition reflex pathway, which have not yet been clarified. The spinal cord and brainstem are essential for the micturition reflex. When the bladder is expanded, bladder-filling information is conveyed by pelvic nerve afferents to the lumbosacral spinal cord segments in rats and then ascends to the brain. In the cerebral cortex, the signal produces the urge to void, while it activates the micturition center [the pontine micturition center (PMC)] in the brainstem. The integrated signal, a command for micturition, descends from the PMC to the lumbosacral spinal cord, which excites the pelvic nerve efferent fibers that produce bladder contraction [10–12]. Contraction increases bladder pressure, which increases afferent nerve discharge, leading to further excitation of PMC neurons and hence producing stronger contraction [13]. Thus, there is positive feedback between the bladder and the PMC. This mechanism makes bladder contraction strong and long enough to void all urine [13]. Perineal stimulation would shut down this positive feedback at the spinal cord and/or brain. Because a segmental somatic tissue (i.e., the perineal area) is the most effective stimulation site among various skin areas, there has been speculation that the effect is mediated in the lumbosacral cord [3, 8].

The aim of this study was to clarify where in the central nervous system (CNS) gentle perineal stimulation inhibits the micturition reflex. For this purpose, we examined whether intrathecally administration of naloxone (around the lumbosacral cord or brainstem) would disinhibit the inhibitory effect of perineal rolling stimulation on the RMC. Then, to determine whether perineal rolling stimulation inhibits descending transmission at the spinal cord, we induced bladder contraction by electrical stimulation of the PMC or descending tract and examined whether the evoked contractions were inhibited by rolling stimulation. To determine whether rolling stimulation inhibits ascending transmission of the micturition reflex at the spinal level, we distended the bladder of animals that were acutely spinalized at the thoracic level, increased the blood flow of the lumbosacral cord, which is an indicator of neuronal activity, and examined whether rolling stimulation would reduce blood flow. We also recorded pelvic nerve afferent-evoked local field potentials in the lumbosacral dorsal commissure, which contains ascending neurons projecting directly to the PMC, and examined whether rolling stimulation would inhibit the local field potentials. The results suggested that the opioidergic system in the lumbosacral cord is involved in the inhibitory effect of perineal rolling stimulation on the RMC, and that this rolling stimulation inhibits both descending and ascending micturition pathways at the lumbosacral level. Some of the present results have been reported in abstract form [14].

## Materials and Methods

The experiments were performed in 29 adult (5–9 months of age) male Wistar rats (offspring of Slc: Wistar rats; body weight 300–420 g). Animals were bred at the Tokyo Metropolitan Institute of Gerontology. The 29 rats were used for six different experiments, to evaluate the effects of perineal stimulation on the activities of the bladder (exp. 1–3), spinal cord (exp. 4–5), or PMC (exp. 6), as summarized in Table 1. A completed ARRIVE guidelines checklist appears in S1 ARRIVE Checklist.

**Table 1:** Summary of the six experimental protocols

	recording		conditions		n
exp. 1 (Fig 1)	bladder	spontaneous RMCs	bladder distension (isometric)	naloxone i.t.	5 (5)
				naloxone i.c.m.	6 (4)
exp. 2 (Fig 2)	bladder	CNS stim.-induced reflex contraction	PMC electrical stim.	-	4 (2)
exp. 3 (Fig 3)	bladder	CNS stim.-induced direct contraction	PMC† or DLF* electrical stim.	hypogastric nerve intact	6 (4)
				hypogastric nerve cut	4 (4)
exp. 4 (Fig 4)	spinal cord	blood flow	bladder distension* (isotonic)	-	7 (5)
exp. 5 (Fig 5)	spinal cord	local field potential	pelvic afferent nerve electrical stim.*	-	8 (4)
exp. 6 (Fig 6)	PMC	multiunit discharge	bladder distension (isotonic)	-	4 (2)

†: deeply anesthetized to avoid production of reflex contraction

*: under acute spinalization.

Numbers before and in parenthesis indicate the number of trials (skin stimulation) and rats, respectively. stim = stimulation

### Ethics statement

This study was conducted in accordance with Guidelines for Proper Conduct of Animal Experiments (established by Science Council of Japan in 2006) and was approved by the animal care and use committee of Tokyo Metropolitan Institute of Gerontology. Animals were housed in groups of two or three per cage with paper or wooden chip bedding in specific pathogen free environment. They had free access to a commercial pelleted diet (CRF-1, Oriental Yeast Co., Ltd., Tokyo, Japan) and tap water with 2 ppm of chlorine. The vivarium was maintained at 22 ± 1°C and 55 ± 5% of relative humidity with a 12-hour light cycle (lights off at 20:00 h). Data collection was conducted in our laboratory between 14:00 and 20:00h.

### General surgery

All surgery and data collection were performed under urethane anesthesia. An advantage of urethane anesthesia is to maintain a stable anesthetized condition during experiment. Basic preparation, including anesthesia and artificial respiration, and recordings of intravesical pressure, blood pressure, and heart rate were essentially the same as in a previous study [8]. Animals were anesthetized with urethane (initial dose 0.9–1.2 g/kg, usually subcutaneously (s.c.) and in some cases intraperitoneally (i.p.)), after initial inhalation of 3% halothane for approximately 3 min. Ventilation was monitored with a gas analyzer (Microcap, Oridion Medical, Jerusalem, Israel), and end-tidal CO_2_ level was maintained at approximately 3.0%. Body temperature was kept at 37°C–38°C using an automatically regulated heating pad and lamp (ATB-1100, Nihon Kohden, Tokyo). A jugular vein was catheterized for intravenous (i.v.) administration of supplemental anesthetics and other drugs. A common carotid artery was catheterized to record arterial blood pressure and heart rate. Animals were artificially ventilated via a tracheal cannula. The depth of anesthesia was routinely judged by observing the animal’s motion, respiration, blood pressure, and heart rate. When these conditions were unstable, 0.4%–1.0% halothane was inhaled during surgery, or additional doses of urethane (0.1–0.15 g/kg s.c. or i.v.) were administered during the experiment. In experiments involving electrical stimulation of neural substrates or recordings of neuronal activities, gallamine triethiodide (20 mg/kg, i.v.) was administered to avoid contamination of skeletal muscle activity. Rats were euthanized by injecting an overdose of pentobarbital at the end of each experiment.

### Recording intravesical pressure

Laparotomy was performed, and a catheter was inserted into the bladder via the anterior urethra. The catheter was secured to the urethra by a thread, closing the urethral cavity in all experiments. To measure intravesical pressure, the urethral catheter was connected to a transducer (TP-200T, Nihon Kohden, Tokyo) via a T-shaped connector. The other end of the T-shaped connector was connected to either a syringe pump or a reservoir to manipulate bladder volume (see below).

### Rhythmic micturition contractions

RMCs were recorded in nine rats. The bladder was filled with saline at a speed of 0.1 ml/min by a syringe pump (EP-70, EICOM, Kyoto) connected to the bladder cannula. The saline infusion was stopped when RMCs (two or three consecutive contractions) were produced. Then, the RMCs continued because the urethra had been closed to keep the bladder volume at a suitable range for the micturition reflex [6, 8].

### Distension of the bladder to a constant pressure

When investigating PMC activity and spinal cord blood flow, distension of the bladder to a constant pressure was accomplished by changing the height of a saline-filled reservoir attached to the bladder cannula. The bladder was abruptly distended for 30–60 s from 0 mmH_2_O to a constant pressure of 100–600 mmH_2_O, which is within the range of amplitude of bladder pressure during RMCs. Bladder distension was repeated at constant intervals of 3–4 min.

### Stimulation of and recording from the PMC

Animals (*n* = 6) were mounted in a prone position on a stereotaxic instrument (SR-5; Narishige, Tokyo). After partial craniotomy, a tungsten electrode was inserted into a unilateral PMC (Barrington’s nucleus [15, 16]), on either the right or the left side. The tip of the electrode was typically located 9.2 mm posterior to the bregma, 1.0 mm lateral to the midline, and 7.0 mm ventral to the surface of the bregma. At the end of each experiment, direct current (100–200 μA) was applied for 30 s to localize the stimulated or recorded site. Then, rats were euthanized by injection of an overdose of pentobarbital, and the brain was removed. Histological verification of the position of the electrode tip was carried out using frozen transverse sections, each 40 μm thick.

#### Stimulation

Electrical stimulation was applied with a stimulator (SEN-7103, isolator; SS-201J, Nihon Kohden) using rectangular current pulses (50–100 Hz, 50–100 μA, 0.2–0.3-ms pulse duration, 150–1000 train pulses). Bladder volume was maintained below the threshold for producing RMCs.

#### Recording

Multiunit discharges were recorded from the PMC with a monopolar electrode (resistance approximately 1 MΩ) using an AC preamplifier (MEG-2100, Nihon Kohden, high cut 3 kHz, low cut 300 Hz). Its output was digitized, and the discharge rates were determined.

### Stimulation of the dorsolateral funiculus

In five rats whose spinal cord was acutely transected at thoracic level (see ‘Spinal transection‘), a coaxial metal electrode (0.1-mm outer diameter; USK-10, Unique Medical, Tokyo) or a tungsten electrode was placed on the surface of the dorsolateral funiculus (DLF) of the thoracic spinal cord at least two segments below the transection, on either the right or the left side. The optimal position for evoking bladder contraction was systematically examined by stimulating the cord with 2–30 train pulses (200 Hz, 200–1000 μA, 0.15-ms pulse duration). Bladder contraction with latency of <2 s was evoked by stimulation as described earlier [17]. Bilateral transection of hypogastric nerves was performed before experiment in three rats. In another rat, hypogastric nerves were transected during experiment, and effect of perineal stimulation was examined both before and after hypogastric nerve transection.

### Measurement of blood flow in the dorsal spinal cord

Blood flow in the spinal cord was recorded, and the response to bladder distension was examined in five rats whose spinal cord was transected at thoracic level. In rats, pelvic afferent neurons locates in the L6 and S1 dorsal root ganglion and terminates at the L3–S3 level of the spinal cord [18]. The animal’s spine was fixed on a stereotaxic instrument (STS-B, Narishige). Laminectomy of the L1 vertebra was performed to expose the dorsal surface of the L5–S1 spinal cord [19]. Blood flow in the dorsal spinal cord was measured with a laser speckle flowmeter (Moor Instruments, Axminster, UK) to find the area where the largest response was observed during bladder distension. Blood flow images were acquired and analyzed as described earlier [20]. Then, the probe (0.5–1-mm diameter; Type N, Advance Co., Tokyo) of the laser Doppler flowmeter (ALF 21, Advance Co.) was placed on the area where the largest response was observed by laser speckle imaging, usually just lateral to the medial vein. The ganglionic blocker hexamethonium was injected (20 mg/kg, i.v.) to prevent possible contamination of autonomic nerve activity influencing the bladder contraction, blood vessels, and therefore the blood flow response [21]. At the end of the experiment, the spinal level of the recording site was confirmed by tracing the spinal nerve roots from the L6 dorsal root ganglion.

### Recording of local field potential in the spinal cord

Using four rats in which the spinal cord was transected at a thoracic level, we recorded local field potentials from the L5–S1 spinal cord that were evoked by electrical stimulation of a pelvic afferent nerve. With the rat in a supine position, the right pelvic nerve bundles were separated from the surrounding tissues and ligated close to the junction of the ureter and bladder. Two silver wire electrodes for stimulation were attached to the central segment of the nerve. Electrodes and the nerve between them were shielded with fixative silicon glue. Then, the rat was turned to a prone position, and the spine was fixed on the stereotaxic instrument. After partial laminectomy of the L1 vertebrae, the tungsten metal electrode (resistance approximately 300 kΩ) was inserted into the dorsal commissure of the spinal cord. A reference electrode was inserted into the paravertebral muscle. Local field potential was recorded using an AC preamplifier (low cut 0.5–5 Hz, high cut 1 kHz). Evoked potentials were elicited by single-pulse electrical stimulation (0.5-ms duration, repeated every 3 s) of the pelvic afferent nerve. Signals were averaged 30 times by a computer. To avoid the influence of changes in afferent nerve activities of intact pelvic fibers (left side), vesical pressure was kept at zero by evacuating urine via a catheter inserted to the bladder, and hexamethonium was injected (i.v.). At the end of each experiment, direct current (50–100 μA) was applied for 30–60 s to localize the recorded site. Then, rats were euthanized by injection of an overdose of pentobarbital. After the spinal level of the recording site was confirmed by tracing the spinal nerve roots from the L6 dorsal root ganglion, the spinal cord was removed, and the tip position of the recording electrode was verified histologically using frozen transverse sections, each 40 μm thick.

### Cutaneous stimulation

Cutaneous stimuli were applied to the skin of the perineum using a roller having a smooth, soft surface made of elastic polymer (Somaplane, Toyoresin Co., Shizuoka; 17 mm in diameter, 15 mm in length, weighing 4 g) [8]. The hair of the stimulated skin area was trimmed with a conventional clipper. The stimulus was applied for a period of 1 min with a rolling speed of approximately 3 mm/s with a frequency of 10 strokes/min. Rolling was performed manually with a force (roller weight) of 4 g and was paced with an auditory cue. Skin stimulation began when recording parameters (the bladder contractions, responses in neuronal activity, and blood flow during bladder distension, or evoked potentials) were stable. We waited for at least 8 min after the end of the skin stimulus to initiate the next trial.

### Spinal transection

Full acute transection of the spinal cord was performed in 14 of the 29 anesthetized rats, at thoracic level. The transection was performed at the upper thoracic level to stimulate thoracic DLF at least two segments below, whereas the spinal cord was transected at the lower thoracic level to record responses in segments L5–S1. Systolic blood pressure was kept above 70 mmHg by i.v. injection of 4% Ficoll 70 (Pharmacia Biotech, Sweden) after spinalization. Data collection was performed 2–5 h after spinalization. After the end of each experiment, the spinal transection was verified visually under microscope.

### Central administration of naloxone

Naloxone hydrochloride (Sigma, USA) dissolved in saline was administered intrathecally (i.t.) into the sacral spinal cord in five rats or into the subarachnoid space of the cisterna magna (i.c.m.) in four rats. For i.t. injection, a catheter (inner diameter 0.2 mm; SP-8, Natsume Co., Tokyo) was inserted through a slit in the atlanto-occipital membrane into the spinal subarachnoid space approximately 10 cm from the slit; the tip of the catheter was placed at the level of the lumbosacral spinal cord. For i.c.m. injection, the catheter was inserted approximately 2–5 mm rostral to a slit in the atlanto-occipital membrane [22]. Naloxone at doses of 1–2 μg (i.t.) was effective in antagonizing the changes in RMCs induced by 10 μg (i.t.) of morphine in urethane-anesthetized rats [23]. Therefore, we used 2 and 20 μg of naloxone in 5 μl of saline. The same volume of saline was used as the control. The order of naloxone and saline was randomized. Each injection was followed by injection of a similar volume of air to flush the dead space in the catheter. The total injection time was > 1 min.

### Sample size

The sample size was based on our previous study on the effect of perineal skin stimulation on RMCs that decreased by 1.45 ± 0.71 times/min (mean ± standard deviation) [8]. With 90% power and a two-sided significance level of 5%, the required sample size was 4, using equations [24] and a software (G*Power 3.1 [25]).

### Data acquisition and analysis

All analog signals obtained (including intravesical pressure, neuronal potentials, and blood flow) were digitized (Micro 1401, Cambridge Electronic Design, UK), for display on a computer monitor and for on-line and off-line analysis using Spike 2 software (Cambridge Electronic Design). Values obtained after the onset of skin stimulation were compared with values obtained before skin stimulation (the pretest-posttest design). Values are expressed as mean ± standard deviation. Changes in RMCs induced by skin stimulation were assessed by one-way repeated measures analysis of variance (ANOVA) followed by Fisher’s least significant difference test. Two-way ANOVA with repeated measures at different time points was performed to compare the changes among saline and naloxone conditions and among intact and transected conditions of hypogastric nerves. Changes in other parameters induced by skin stimulation were assessed by one-way ANOVA followed by Fisher’s least significant difference test. Statistical significance was set at the 5% level. One or two trials in each rat were used for the statistical analysis.

## Results

### Effect of central administration of naloxone on RMCs that were inhibited by skin stimulation

**Fig 1:**
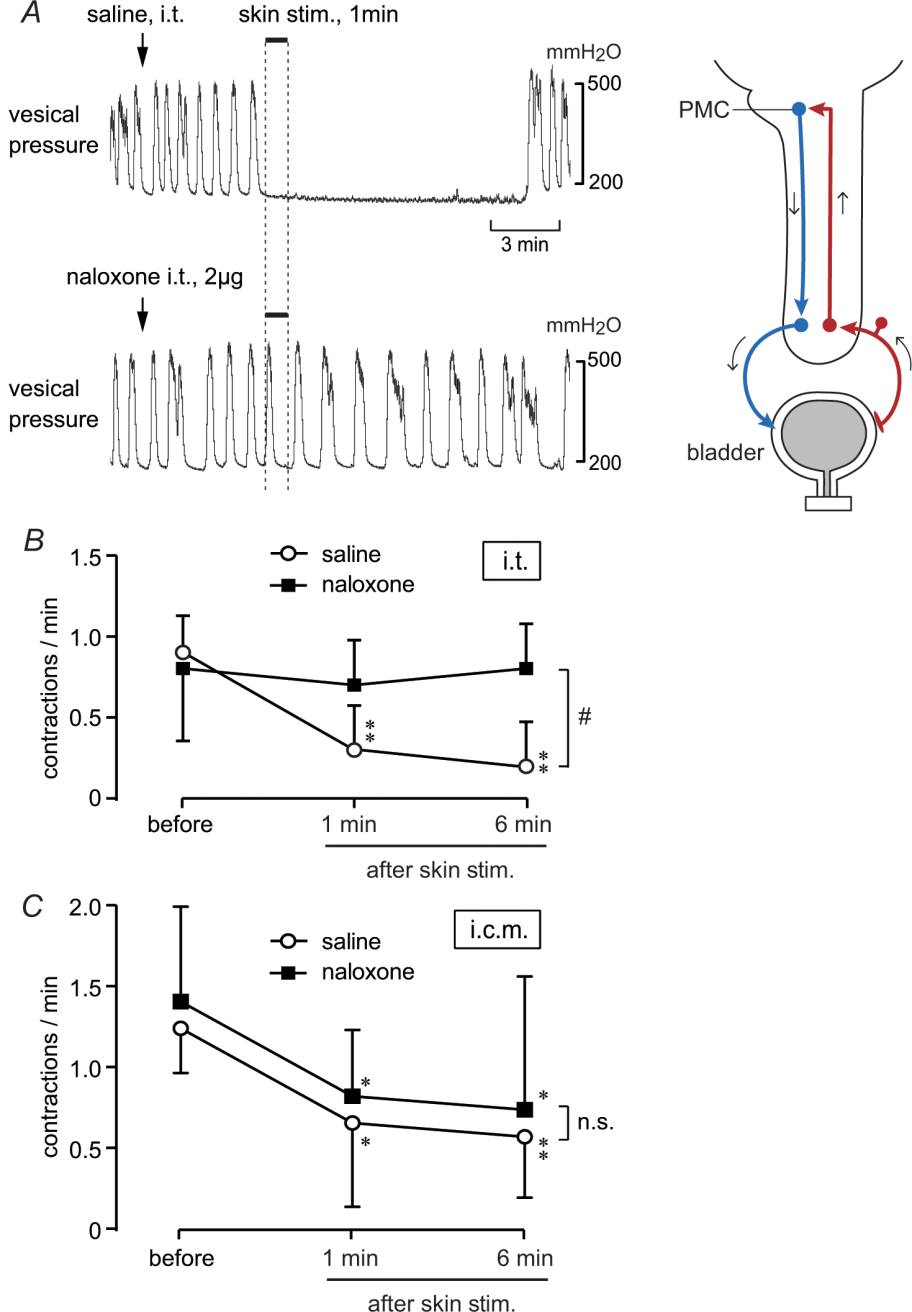
Effects of i.t. and i.c.m. administration of naloxone on inhibition of rhythmic micturition contractions (RMCs) by rolling stimulation. (A) Examples of the effects of i.t. saline (upper trace) and i.t. naloxone (2 μg, lower trace) administration in the same rat. Rolling stimulation applied to the perineal area for 1 min is indicated by the upper bar and vertical dotted lines. Summarized graph of (B: *n* = 5 from five rats) i.t. and (C: *n* = 6 from four rats) i.c.m. saline (white circles) and naloxone (2–20 μg, black squares) administration. Contractions were counted before 1 min (-2–0 min), 1 min (0–2 min), and 6 min (5–7 min) after the onset of skin stimulation, and expressed as frequencies per minute. *p < 0.05, **p < 0.01; significantly different from the corresponding value before skin stimulation (one-way repeated measures ANOVA followed by Fisher’s least significant difference test). #*p* < 0.05 (two-way repeated measures ANOVA).

The effect of perineal stimulation on RMCs after i.t. administration of the opioid receptor antagonist naloxone, in comparison with saline, was examined in five trials in five rats to define the role of spinal opioids in mediating the perineal stimulus-induced inhibition of RMCs. In a saline-injected rat shown in Fig 1A upper trace, stimulation of the perineal skin with a roller inhibited RMCs completely for more than 10 min. Subsequently, the RMCs recovered. Complete inhibition was observed in another individual rat. In the remaining three rats, perineal rolling stimulation gradually reduced the frequency of RMCs. Amplitudes of recovered RMCs were almost the same as those before skin stimulation in all five rats. In contrast, frequencies of RMCs were lower than those before stimulation. The frequency of RMCs was 0.90 ± 0.22/min before stimulation and significantly decreased to 0.30 ± 0.27/min and 0.20 ± 0.27/min at 1 and 6 min after stimulation, respectively (Fig 1B, white circles; *p* < 0.01 by one-way repeated ANOVA). The effects of perineal stimulation were similar to those reported without saline injection [8, 9]. Intrathecal injection of naloxone neither affected the basal pressure between the RMCs nor the frequency and amplitude of RMCs (lower trace in Fig 1A). Naloxone did not produce any excitatory effect on the bladder in all rats tested, as previous reports on naloxone i.t. in rats [26, 27]. However, perineal stimulation reduced the frequency of RMCs only slightly, in contrast to the marked reduction that was observed in the control (i.t. saline administration; compare the two traces in Fig 1A). Inhibition of RMCs by perineal stimulation was almost abolished in all rats tested after i.t. administration of naloxone (2 μg, *n* = 2; 20 μg, *n* = 3). Naloxone (i.t.) significantly reduced the inhibitory effect of cutaneous stimulation (*p <* 0.05, two-way repeated ANOVA) compared with i.t. saline (Fig 1B). The influence of naloxone persisted for 20 min at 2 μg and for more than 60 min at 20 μg.

We also administered naloxone i.c.m. in six trials in four rats, to exclude the possibility that the effect of i.t. administration of naloxone was due to the spread of naloxone into the brainstem. Naloxone (i.c.m., 2 μg, *n* = 3; 20 μg, *n* = 3; the same doses as those used for i.t. injection) did not affect either baseline RMCs or the inhibitory effect of cutaneous stimulation on RMC frequency. There were no significant differences in the frequency of RMCs between naloxone and saline administration (Fig 1C).

### Bladder contraction induced by central stimulation

Bladder contractions induced by electrical stimulation of the PMC were examined in four rats. Electrical stimulation of the PMC was reported to elicit two types of bladder responses: (1) small-amplitude short-duration responses due to direct activation of the descending excitatory pathway from the PMC to the sacral parasympathetic nucleus (induced-direct contractions) and (2) large-amplitude long-duration reflex responses induced by the direct contractions but maintained by afferent feedback (induced-reflex contractions) [28].

**Fig 2:**
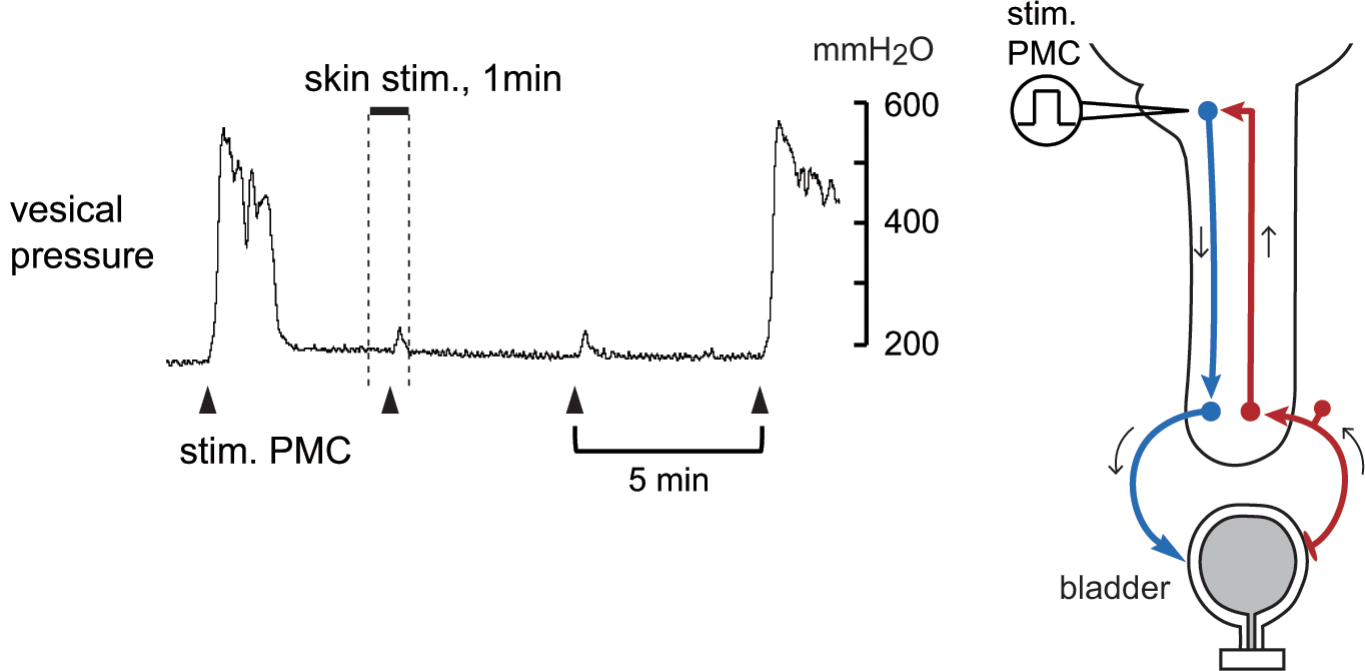
Pontine micturition center (PMC) stimulation-induced reflex bladder contractions, before, during, and after rolling stimulation. Rolling stimulation applied to the perineal area for 1 min is indicated by the upper bar and vertical dotted lines. Bladder contractions induced by electrical stimulation of the PMC (100 Hz, 50 μA, 0.2-ms pulse duration, 1000 train pulses every 5 min) in a rat. Note that induced reflex contractions with large amplitude were inhibited during and 5 min after rolling stimulation. Instead, induced direct contractions with a small amplitude [28] were observed.

First, we confirmed that repetitive electrical stimulation of the PMC (0.2 ms, 50 μA, 100 Hz, 1000 pulses) produced a marked increase in bladder pressure (i.e., induced-reflex contractions), similar to micturition contraction. When perineal rolling stimulation was applied, the amplitude and duration of PMC stimulation-induced reflex contraction were much reduced (Fig 2). Instead, small-amplitude short-lasting contractions (i.e., induced-direct contractions) were induced. Among four trials tested in two rats, remarkable inhibition of the reflex contraction was observed in two trials. In the remaining two trials, the perineal rolling stimulation partially reduced the amplitude of contractions by 40%–70%. Inhibition of induced-reflex contraction lasted for 6–15 min before recovery.

**Fig 3:**
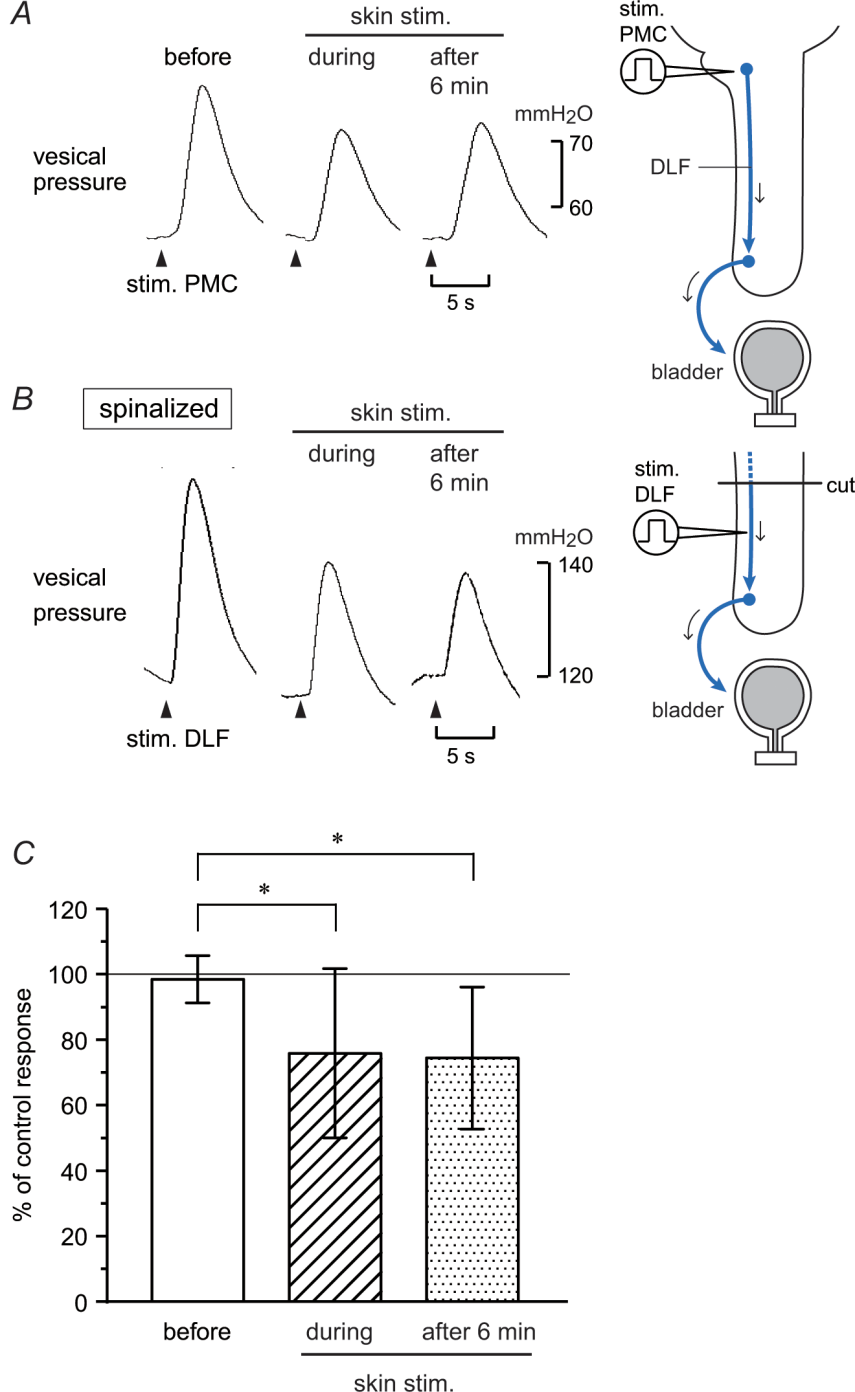
Electrical stimulation-induced direct contractions of the bladder, before, during, and after rolling stimulation. (A) Direct contractions induced by electrical stimulation of the pontine micturition center (PMC; 50 Hz, 100 μA, 0.3-ms pulse duration, 150 train pulses every 1 min) under low vesical pressure in a deeply anesthetized rat. (B) Dorsolateral funiculus (DLF) stimulation-induced direct contractions of the bladder in a rat acutely spinalized at the upper thoracic level. Stimulation (200 Hz, 500 μA, 0.15-ms pulse duration, two pulses every 1 min) was applied to the DLF at T7. (C) Summary (*n* = 6 from four rats) of the magnitude of direct contraction induced by electrical stimulation of either the PMC or the DLF. Values are expressed as percentages of the mean of two control responses obtained before skin stimulation. Peak changes within 5 s after PMC or DLF stimulation were measured. *p < 0.05 (one-way ANOVA followed by Fisher’s least significant difference test).

In two rats under deeper anesthesia (initially anesthetized with urethane at 1.2 g/kg i.p., according to [15]), repetitive electrical stimulation of the PMC produced induced-direct contractions with a small amplitude and of a shorter duration. We examined the effect of perineal rolling stimulation on induced-direct contractions. Skin stimulation slightly reduced the amplitude of contractions during and 6 min after perineal stimulation (Fig 3A). Inhibition lasted for 8 min before recovery. Similar results were obtained in all four trials in two rats. To confirm the possibility that the inhibition of induced-direct contractions by skin stimulation occurred at the spinal level, we induced direct contractions by stimulation of the descending tract from the PMC. In rats whose spinal cord was transected at thoracic level, the DLF caudal to the transection level was stimulated. Perineal rolling stimulation partially reduced the amplitude of DLF stimulation-induced bladder contractions (Fig 3B). The reduction was similar in magnitude and time course to the reduction in direct contractions caused by PMC stimulation. Similar results were obtained in two trials in two rats. The amplitude of direct contractions induced either by PMC stimulation or by DLF stimulation (a total of six trials in four rats) decreased significantly (*p* < 0.05) to 76% ± 26% and 74% ± 22% of control responses during and 6 min after skin stimulation, respectively (Fig 3C). The perineal rolling stimulation did not produce any excitatory effects on the bladder of either spinal cord-intact or transected animals.

Contribution of hypogastric nerve to the effect of perineal rolling stimulation on induced-direct contractions was examined by bilateral transection of the hypogastric nerves in four rats. Perineal rolling stimulation reduced the amplitude of DLF stimulation-induced bladder contractions to 68% ± 27% and 88% ± 6% of control responses during and 6 min after skin stimulation, respectively. There were no significant differences in magnitude of inhibition of direct contraction between hypogastric nerve intact (n = 6) and transected (n = 4) conditions (by two-way ANOVA).

### Lumbosacral dorsal spinal cord responses induced by afferent inputs from the bladder

Inputs from the bladder afferents to spinal ascending neurons are essential to induce the micturition reflex. To assess whether this transmission would be inhibited by perineal rolling stimulation, we examined blood flow and neuronal responses in the lumbosacral dorsal spinal cord.

**Fig 4:**
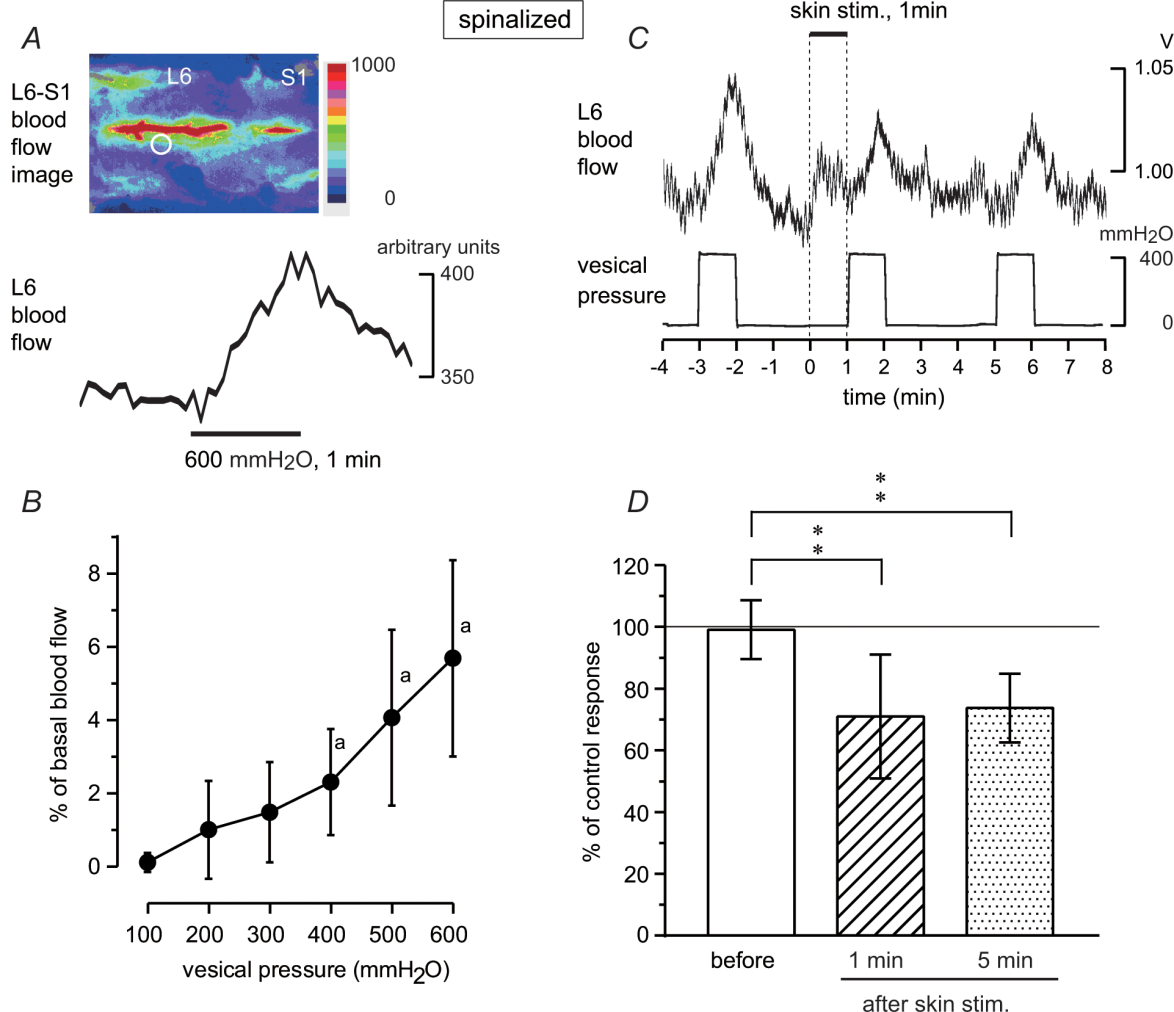
Changes in lumbosacral dorsal spinal cord blood flow during isotonic bladder distension in acutely spinalized rats. (A) Representative example of changes in blood flow measured by laser speckle flowmetry in a rat. Narrow red areas correspond to the central vein. The white circle indicates where blood flow changes were measured (A) with a laser speckle flowmeter and (B–D) a laser Doppler flowmeter. (B) Summary (*n* = 5 from five rats) of blood flow changes during isotonic bladder distension to different pressure, expressed as percentages of basal blood flow. a, *p* < 0.05; significant difference determined by one-way repeated ANOVA followed by Fisher’s least significant difference test. (C, D) Effect of perineal rolling stimulation on blood flow responses during bladder distension. (C) Upper trace: example of blood flow changes. Lower trace: vesical pressure. Rolling stimulation for 1 min is indicated by the upper bar and vertical dotted line. (D) Summary (*n* = 7 from five rats) of the magnitude of blood flow response, expressed as a percentage of the mean of two control responses obtained before skin stimulation. Peak changes during bladder distension relative to the basal flow at the onset of distension were measured. **p < 0.01 (one-way ANOVA followed by Fisher’s least significant difference test).

Blood flow in the L5–S1 dorsal spinal cord was recorded in five rats acutely spinalized at thoracic level, and changes in blood flow during isotonic bladder distension were measured by laser speckle imaging and laser Doppler flowmetry. The penetration depth of the tissue is approximately 1 mm (by laser Doppler flowmeter) or less (by laser speckle imaging), which covers the dorsal part of the spinal cord. Real-time imaging of blood flow revealed that the largest response was observed during bladder distension at the entry zone of the L6 dorsal root around the central vein (see image in Fig 4A). When the bladder was distended by applying a constant pressure of 600 mmH_2_O for 1 min, blood flow increased gradually and reached its maximum at the end of stimulation. The increased blood flow gradually returned to the basal level after the end of distension (a trace in Fig 4A). Then, the probe of the laser Doppler flowmeter was placed on the area where the largest response was observed by laser speckle imaging (circle in the image in Fig 4A). Responses measured by laser Doppler flowmetry were similar to those measured by laser speckle imaging. Bladder distension to a constant pressure of 200–600 mmH_2_O produced increases in blood flow during distension, depending on pressure (Fig 4B). Significant responses were obtained with pressures above 400 mmH_2_O. We examined the effect of perineal stimulation on the blood flow response produced by constant pressure stimulation. During perineal rolling stimulation, a slight and transient increase in blood flow was observed. However, following skin stimulation the response to bladder distension was reduced (Fig 4C). The inhibitory effect was still observed 5 min after skin stimulation. The results obtained in seven trials in five rats, using constant pressure stimuli of 400–500 mmH_2_O, are summarized in Fig 4D. The blood flow response during bladder distension was significantly reduced to 71% ± 20% (*p* < 0.01) and 74% ± 11% (*p* < 0.01) of control responses 1 and 5 min after skin stimulation, respectively.

**Fig 5:**
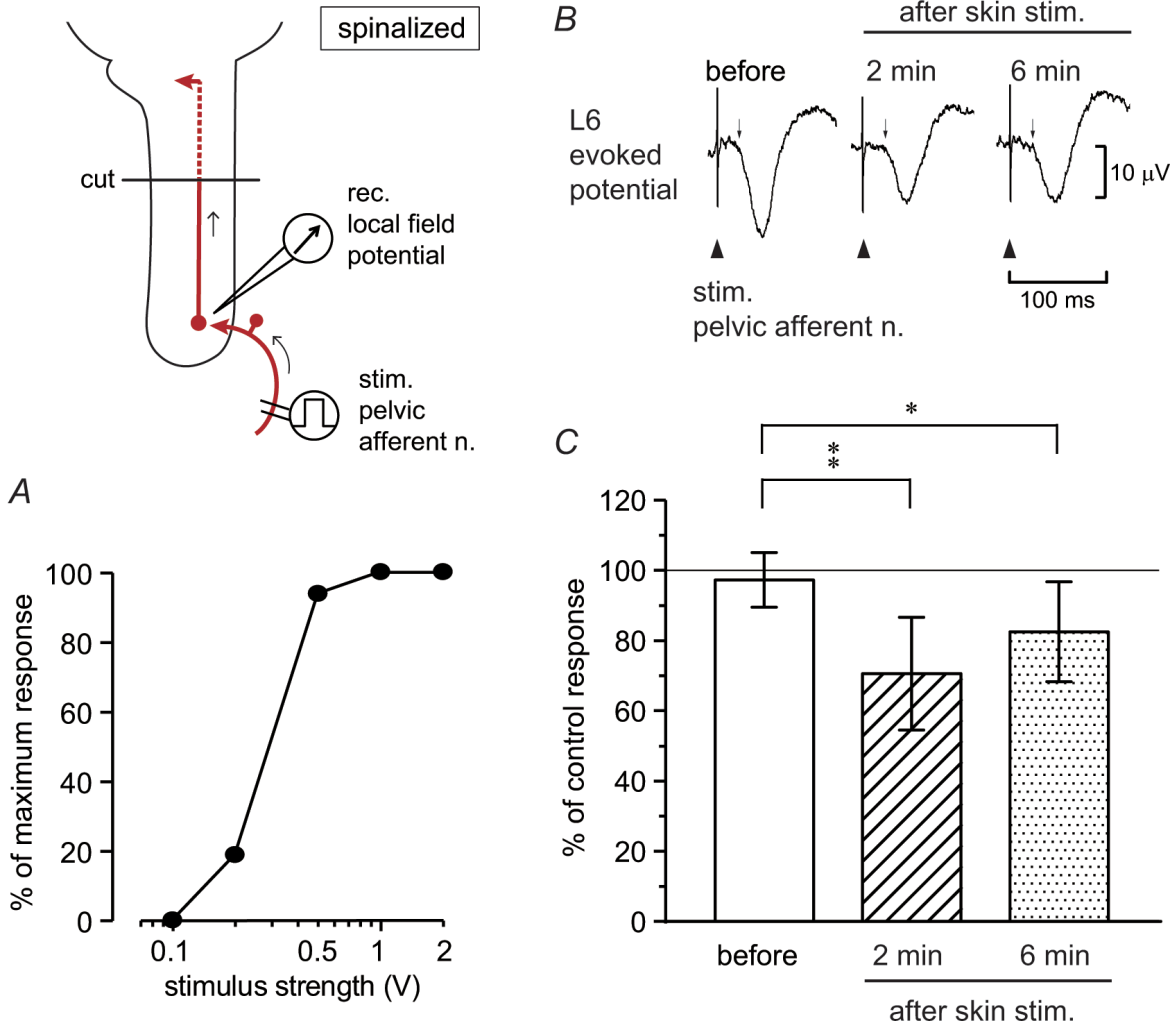
Evoked potentials in lumbosacral dorsal commissure after stimulation of the pelvic afferent nerve in acutely spinalized rats. The pelvic afferent nerve was stimulated with a single impulse (0.5 ms, every 3 s) and the evoked responses were averaged 30 times. (A) Plots of the relation between stimulus strength (abscissa) and the size of the potential, expressed as the percentage of maximum size in a rat. (B, C) Effect of perineal rolling stimulation on the lumbosacral potential. Averaged responses before or 2 min (1–3 min) and 6 min (5–7 min) after the onset of skin stimulation. (B) Example in a rat. (C) Summary (*n* = 8 from four rats) of the magnitude of the evoked potential, expressed as a percentage of the mean of two control potentials obtained before skin stimulation. Peak changes within 100 ms after stimulation of the pelvic nerve (at 1 V) were measured. **p < 0.01, *p < 0.05 (one-way ANOVA followed by Fisher’s least significant difference test).

The vesical pelvic afferent nerve was electrically stimulated close to the bladder, and evoked potentials were recorded from the dorsal commissure of the lumbosacral spinal cord at L5–S1 levels in four acutely spinalized rats. Single electrical stimulation (0.2–2 V) evoked a field potential with a latency of approximately 20 ms. The amplitude of the evoked potential increased up to 1 V of stimulation (Fig 5A). We set the strength of electrical stimulation at 1 V, and perineal rolling stimulation was examined. Rolling stimulation reduced the amplitude of the evoked potential slightly (Fig 5B). Similar results were obtained in all eight trials in four rats. Inhibition lasted for 4–13 min before recovery in each trial. The size of the evoked response, measured at the peak amplitude, was reduced to 70% ± 16% (*p* < 0.01) and 82% ± 14% (*p* < 0.05) of the control response 2 and 6 min after skin stimulation, respectively (Fig 5C).

The inhibitory effects of perineal stimulation on the blood flow response and the evoked potential in the two acutely spinalized rats were abolished after naloxone (2 mg/kg, i.v.).

### Multiunit activity of PMC neurons during isotonic bladder distension

**Fig 6:**
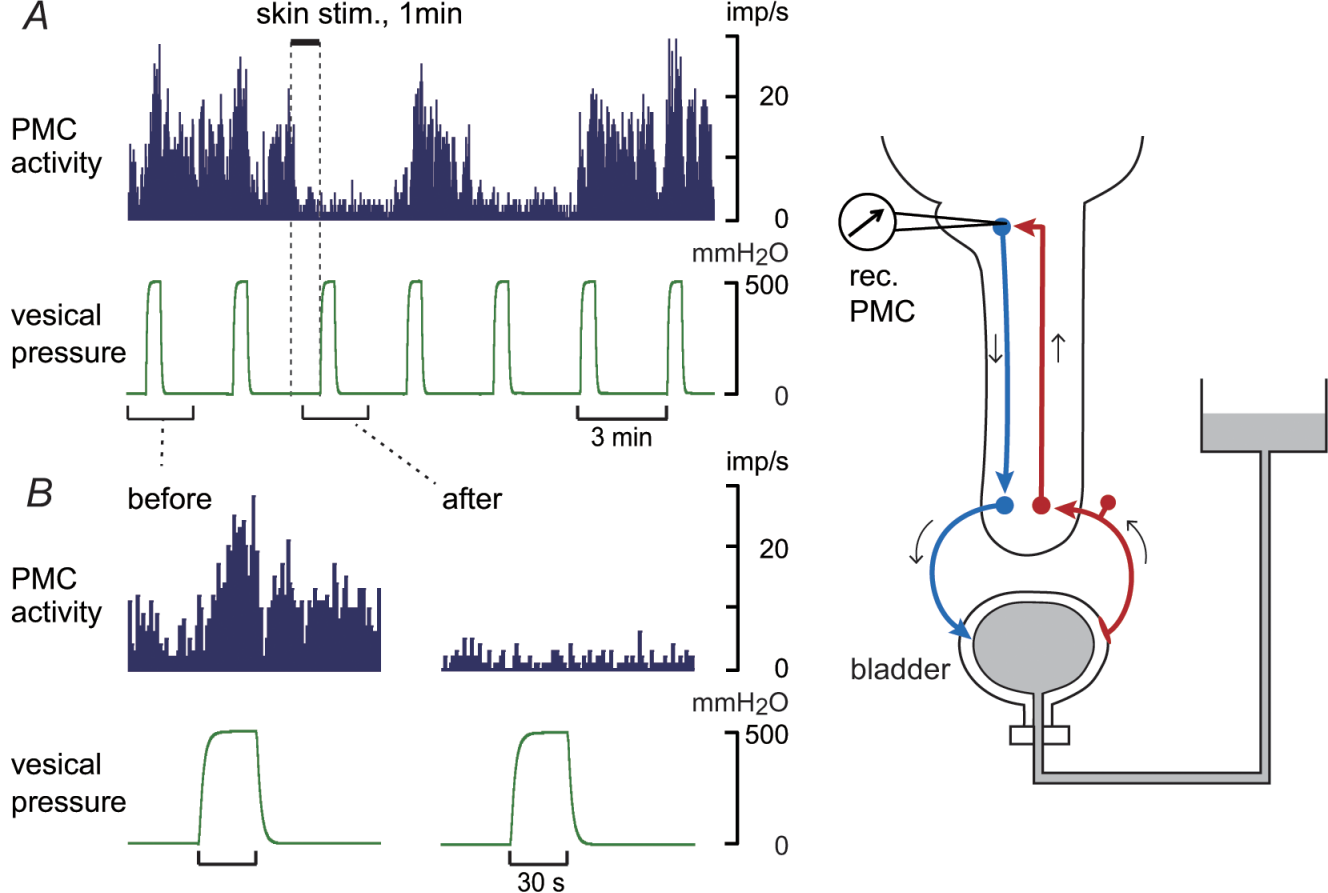
Multiunit activity of pontine micturition center (PMC) neurons before and after rolling stimulation. (A) An example of the effect of perineal skin stimulation on PMC neuron activity. (B) Enlarged views before and after skin stimulation are presented. Upper traces: neural activity determined every 1 s. Lower traces: vesical pressure. The bladder was isotonically distended for 30 s.

The activity of PMC neurons was examined during isotonic distension of the bladder in two rats. Bladder distension (500 mmH_2_O) markedly increased the activity of multiunits in the PMC (Fig 6). However, neuronal activity during bladder distension was reduced after perineal rolling stimulation was applied. The reduction of activity was observed in two distensions out of three distensions in total, tested during 7 min after the start of perineal stimulation. Tonic activity, which was observed when bladder pressure was low, was also decreased. Such a reduction was observed in all four trials tested in two rats when applying constant pressure of 300–500 mmH_2_O; the reduction of activity was observed in 1–3 distensions out of all the three distensions. Inhibition lasted for 10–13 min before recovery. PMC neuronal activity during bladder distension was decreased to 42% ± 23% and 48% ± 43% of the control response at 1 and 7 min after the start of skin stimulation, respectively.

## Discussion

**Fig 7:**
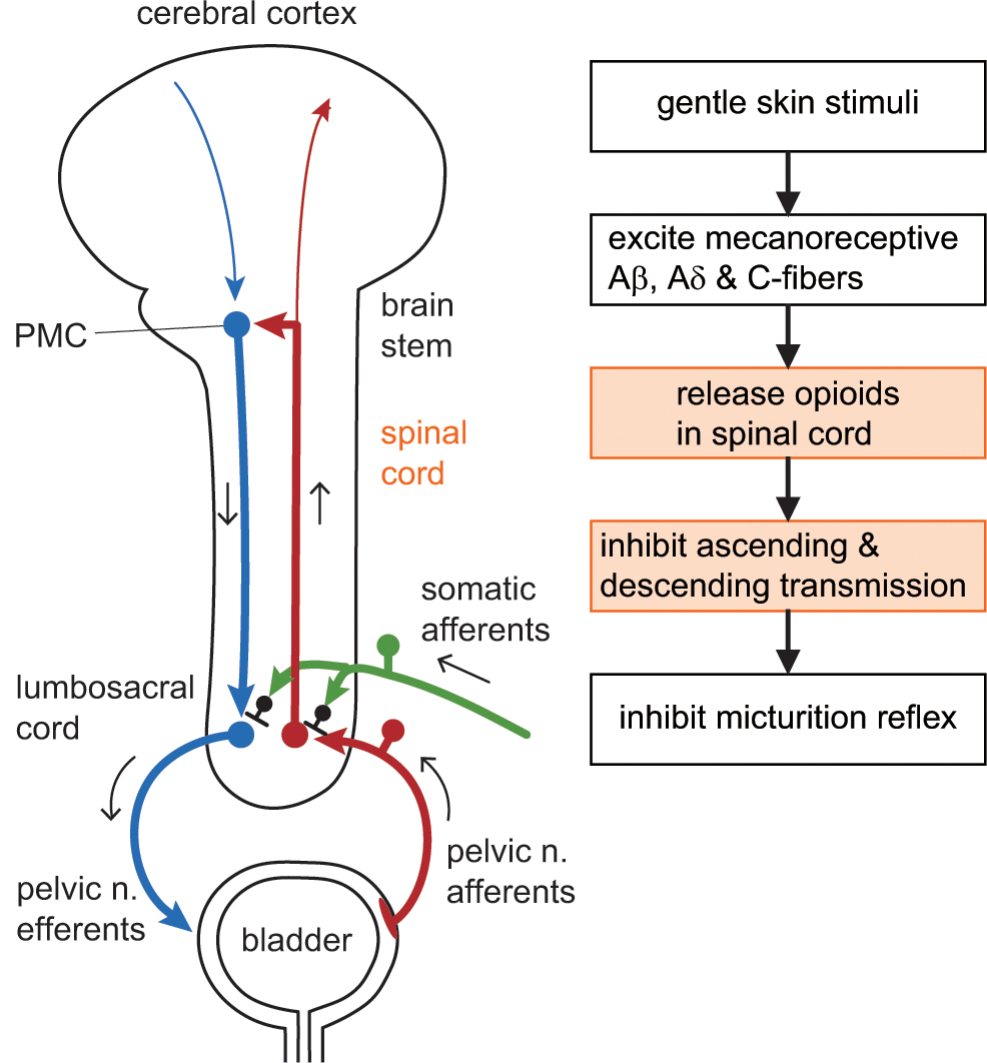
Schema illustrating the mechanisms underlying the effect of gentle perineal stimulation on the micturition reflex pathway. Gentle stimulation of the perineal skin excites low-threshold mechanoreceptive Aβ, Aδ, and C afferent fibers. These fibers activate the spinal opioidergic system and inhibit both ascending and descending transmissions of the micturition reflex pathway at the spinal cord level. These inhibitions would lead to the shutting down of positive feedback between the bladder and pontine micturition center (PMC), resulting in inhibition of the micturition reflex. The hypogastric nerve innervating the bladder is omitted because bilateral transection of hypogastric nerves had no effect on the inhibitory effect by gentle perineal stimulation.

We reproduced our previous studies in which RMCs were inhibited by gentle stimulation of the perineal skin with a soft roller [8, 9]. The present study showed that this inhibition was disinhibited by naloxone administered by the i.t. route but was not affected by the same dose of naloxone administered i.c.m., indicating that the opioid system in the spinal cord mediates this inhibition. Rolling stimulation inhibited the bladder contractions evoked by direct stimulation of the descending pathway, indicating that the location of inhibition of this pathway by perineal rolling stimulation is the lumbosacral cord. The activity of lumbosacral neurons monitored by blood flow or evoked potentials was inhibited by perineal rolling stimulation. Perineal stimulation may inhibit the ascending pathway of the micturition reflex at the lumbosacral level. Fig 7 summarizes the results and conclusions of the present study.

It has been reported that inhibition of RMCs by noxious pinching stimulation of the perineal skin was not affected by total destruction of the hypogastric nerve branches in both rats and cats [3, 5, 29]. Similarly, we showed that bilateral transection of hypogastric nerves had no effect on the inhibition of either spontaneous RMCs [8] or CNS stimulation-induced direct bladder contractions (this study) by gentle perineal stimulation. It is no doubt that parasympathetic pelvic nerves contributes to the effect as an efferent path. However, partial contribution of hypogastric nerve to inhibition of bladder contraction by other kinds of peripheral stimulation was suggested by the fact that hypogastric nerve was activated by vaginal stimulation [30], and that inhibitory effect by pudendal nerve stimulation of spinal reflex bladder activity during noxious bladder distention was partially reduced by propranolol [31]. Although we did not record hypogastric nerve activity, our stimulation appears to be too gentle to evoke sympathetic activation.

Naloxone is an antagonist of μ-, κ-, and δ-opioid receptors [32]. In the sacral spinal cord, enkephalin-like immunoreactivity in nerve processes is most densely concentrated in the superficial dorsal horn, in the intermediate gray matter, and around the central canal [33]. There are direct symmetric synaptic connections between endomorphin 2-containing primary afferent terminals and μ-opioid receptor-expressing neurons in the parasympathetic nucleus [34]. Our recent study also suggested that spinal μ-opioids are involved in the segmental inhibitory mechanism in which noxious heat stimuli-induced cardiac reflexes are inhibited by gentle skin touch [35]. Such an endogenous opioid system in the sacral cord would inhibit the ascending and/or descending limb of the micturition reflex pathway.

We first examined whether perineal rolling stimulation inhibits descending excitatory transmission from the PMC to the sacral bladder preganglionic neurons. Rolling stimulation partially inhibited the direct contraction induced by PMC stimulation (Fig 3A). This stimulation also inhibited the direct contraction induced by DLF stimulation in the acutely spinalized rats (Fig 3B), indicating that the descending transmission of the micturition reflex pathway is inhibited at the spinal cord. It has been shown in cats that neurons in the PMC increase their activity during micturition contraction, project directly to the sacral spinal cord [11, 36], and connect polysynaptically to sacral bladder preganglionic neurons [37]. In rats, bladder preganglionic neurons exist in L6 and S1 [38]. Opioid neurons in the lumbosacral cord may inhibit intercalated neurons, preganglionic neurons, and/or axon terminals of the spinally projecting neurons from the PMC in rats. Inhibition of micturition contraction by perineal stimulation has also been suggested to be primarily due to inhibition of the excitatory input to preganglionic neurons [39].

Next, we examined whether the ascending limb of the micturition reflex pathway in the spinal cord is affected by perineal rolling stimulation. It is known that neurons that respond to bladder distension distribute broadly in the dorsal and middle parts of the lumbosacral cord [40–42]. Interneurons in the dorsal commissure also receive inputs from the vesical primary afferents [12, 43–45]. Using acutely spinalized rats, we examined the activities of spinal neurons that were induced by afferent input from bladder by studying two variables: (1) blood flow change in the dorsal area of the lumbosacral cord in response to bladder distension and (2) local field potentials in the lumbosacral dorsal commissure that were evoked by pelvic afferent nerve stimulation. We found that perineal rolling stimulation inhibited both excitatory responses (blood flow and local field potentials) by approximately 30%, with a similar time course. These inhibitions may occur in several kinds of spinal neurons such as local interneurons, long ascending neurons, and short ascending neurons. Among these neurons, long ascending neurons in the spinal parasympathetic nucleus and in the dorsal commissure project directly to the PMC [46]. Reduction in the firing activities of PMC neurons after perineal rolling stimulation (Fig 6) could be explained by decreased activities of the ascending limb of the micturition reflex pathway. Candidate neurons that may cause this decrease may be long ascending neurons in the spinal parasympathetic nucleus and/or the dorsal commissure; at least the latter neurons were inhibited by perineal stimulation and would convey reduced information to the PMC.

Based on the latency (approximately 20 ms) and the distance between the stimulating electrode and lumbosacral spinal cord (approximately 100 mm), the conduction velocity calculated (5 m/s) was in the same range as for afferent Aδ fibers in the pelvic nerve [47]. Therefore, the evoked potential appears to be induced by the excitation of afferent Aδ fibers in the pelvic nerve. Pelvic nerve afferents are composed of Aδ and C fibers [12, 47]. In CNS-intact animals, the micturition reflex is mediated by Aδ afferents [48, 49], while in chronic spinal animals, C fibers in the vesical afferents of the pelvic nerve evoke bladder contractions [12]. Chemical stimulation of C fibers in the vesical afferents evokes bladder contractions in acute spinal animals [50]. In these preparations, bladder contractions were inhibited by electrical stimulation of the cutaneous nerve [12, 50]. In contrast, we provide here the first evidence that transmission of vesical pelvic Aδ afferents in the sacral cord is also inhibited by somatic afferent stimulation. Because we recorded the evoked potential after acute spinalization but not chronic spinalization, the Aδ potential we observed may relate to the normal micturition reflex in CNS-intact condition.

The areas around the PMC are related to the regulation of sleep and wakefulness [16]. Any peripheral sensory input can be an awakening stimulus. However, in our daily experience gentle skin stimulation can be sedative and facilitate falling asleep. The present results showed that gentle skin stimuli only have an inhibitory effect on PMC activity (Fig 6). In contrast, in the case of acupuncture stimulation, excitation was observed following acupuncture stimulation of sacral vertebrae in some neurons related to bladder activity in the PMC [51]. Similarly, in the low vesical pressure condition in both spinal cord-intact and transected animals, noxious pinching or non-noxious brushing stimulus of the perineal skin has an excitatory effect (i.e., produces bladder contraction) [3]. However, the present gentle perineal stimulation with a soft roller did not produce such an excitatory effect. This gentle stimulation, which produces very low-frequency activity in low-threshold mechanoreceptive Aβ, Aδ, and C fibers [8], appears to have only inhibitory effects on the micturition reflex.

Perineal rolling stimulation only partially suppressed the amplitude of CNS stimulation-induced direct contraction of the bladder in both CNS-intact and spinal cord transected animals (Fig 3). This indicates that descending transmission of the micturition reflex was partially suppressed by approximately 20% at the spinal cord. In contrast, reflex responses, i.e., spontaneous RMCs (Fig 1), PMC stimulation-induced reflex bladder contractions (Fig 2), and bladder distension-induced excitation of the PMC (Fig 6) were reduced by approximately 50% or more. Such a difference in the extent of inhibition may be explained by an additional inhibition of ascending transmission by approximately 30%, which was suggested by our results (Figs 4 and 5). Although we cannot exclude the possibility that supraspinal pathways may also be involved in this type of somatic inhibition on the micturition reflex, the present results clearly demonstrate the significance of the spinal cord as the major site of inhibition by gentle perineal stimulation.

### Clinical significance

Anticholinergic drugs are the most common pharmacological treatments for overactive bladder. Such drugs suppress cholinergic transmission at peripheral sites. However, our results suggest that somatic input affects the micturition reflex in the spinal cord in the CNS but not at peripheral receptors. Rolling with a soft roller on the skin surface appears to be an effective technique for exciting the relevant afferent fibers to produce prolonged segmental inhibition of both ascending and descending transmissions of the micturition reflex pathway in the spinal cord, by activating the spinal opioidergic system. The CNS mechanisms clarified in the present study can explain the results of a recent clinical study on the efficacy of self-care with an elastic roller in nocturia. In that study, gentle perineal stimulation for 1 min applied before going to bed produced a significant reduction in the frequency of nocturia in elderly women with an overactive bladder [52]. The causes of overactive bladder have not clarified, but, theoretically, increased afferent nerve activity, decreased inhibitory control in the CNS, and increased sensitivity of the detrusor to efferent stimulation may be involved [53]. Therefore, based on the CNS mechanisms, we demonstrate here, gentle perineal stimulation may be applicable to several different types of overactive bladder.

## Supporting Information

S1 ARRIVE Checklist Completed “The ARRIVE Guidelines Checklist” for reporting in vivo animal experiments in the present study. (PDF)
